# ‘A team around the child’ professionals’ experiences of unmet needs, access and expectations in children’s palliative care services, a phenomenological study in the UK

**DOI:** 10.1177/13674935221147716

**Published:** 2023-03-22

**Authors:** Georgina Constantinou, Erica J Cook, Elaine Tolliday, Gurch Randhawa

**Affiliations:** 1 Institute for Health Research, 5195 University of Bedfordshire, UK; 2 Centre for Maternal and Child Health Research, School of Health Sciences, 4895 City University of London, London, UK; 3 Department of Psychology, 5195 University of Bedfordshire, UK; 4 58686 Keech Hospice Care, Luton, UK

**Keywords:** Palliative care, hospice and palliative care nursing, pediatrics, hospice care, respite care

## Abstract

This study aims to understand the experiences of professionals involved in caring for families of children with life-limiting illnesses to ascertain unmet needs, access issues and expectations of services.

A phenomenological approach comprising semi-structured interviews with professionals from various services was used. Interviews that happened between July and November 2018 were audio-recorded, transcribed verbatim and thematically analysed based on descriptive phenomenology.

In total, 29 interviews were conducted. Findings showed how essential collaborative working was for becoming a team around the child. Barriers to this include complex working relationships, overprotectiveness of families, roles and responsibilities and use of independent care records. The pressures of waiting times and the impact of staffing shortages affected the experiences of providing care. The reality of meeting families’ expectations was shaped by family networking, online research and previous services resistance, which was influential in more challenging interactions. Expectations of care were also impacted by misunderstandings and anxieties surrounding access to services. Overall, professionals were concerned about families being used as a bottomless caring resource and stressed the need for short breaks to alleviate parents.

Interventions that educate families and professionals on these services and how they can benefit the child and family would be well received.

## Introduction

More than 21 million children worldwide may need children’s palliative care (CPC), with around 8 million needing specialist palliative care services (Connor et al., 2017; Marston et al., 2018). In England, the number of children diagnosed with a life-limiting or life-threatening condition (LLC/LTC) has more than doubled in the past 20 years to 86,625 as of 2017/2018 (Fraser et al., 2020), suggesting a rising need for care that meets the needs of this population (Fraser et al., 2012, 2020). These children could require access to CPC services in their lifetime (National Institute for Care Excellence (NICE), 2016), and families may need input from; healthcare (hospital, hospice, community), social care, education, therapy (occupational, art, music, play and complimentary), spiritualist services and many more due to the complexity of their condition (NICE), 2016). CPC is often misinterpreted by professionals and families as end-of-life care (EOL) (Krau, 2016; Twamley et al., 2014; Maciasz et al., 2013). It is important to note that CPC is a service provided to children and their families with life-limiting conditions, from the point of diagnoses, along the disease trajectory which can span many years before reaching the EOL phase (Together for Short Lives, 2018).

Although palliative care for children is a recognised speciality, it is still relatively new and is establishing and developing internationally (Together for Short Lives (2017a). Evidence suggests that provisions worldwide vary significantly between countries (Knapp et al., 2011). The national and international research priorities for CPC state that we need more evidence which will increase the understanding of children and their families’ needs throughout their ‘lifetime’ of care from diagnosis to bereavement (Baker et al., 2015; Downing et al., 2015), identifying the issues patients, families and professionals face and the circumstances in which they arise (Together for Short Lives, 2018). To address this research priority, it is necessary to explore the experiences of those professionals working to meet families' needs across this ‘lifetime’ of services. This understanding is vital for the development and improvement of services. The literature evidences challenges facing services in the UK, detailing; availability issues reported as a ‘postcode lottery’ with the services a family can often access dependent on where a family lives (Together for Short Lives, 2017b; 2021a) and inconsistent funding and resources (Together for Short Lives, 2021a) which are vital to equip services to meet the needs of families. This issue is predicted to worsen for many CPC services due to COVID-19 (Together for Short Lives, 2021b). This climate adds increased pressure to those professionals with reports of stress, burnout and difficulty with retention (Whiting et al., 2021).

There remains limited understanding of how well palliative care services deliver care that meets the needs of families. Based on families’ experiences, an international scoping review identified that families might have unmet needs (Constantinou et al., 2019) across several areas of the NICE Quality Standards (National Institute for Care Excellence (NICE, 2017). Evidence in this review was found in 17 international locations, with the United Kingdom, United States, Australia, Germany and Canada leading contributors. These studies often focused on a specific service or aspect of care, limiting its generalisability to the broader pathway of services that families will access in their lifetime (Together for Short Lives, 2018).

Professionals’ experiences also suggest that meeting families’ expectations of care can be challenging, with evidence showing it is not always possible to provide the care that would meet expectations (Mack et al., 2020). Influences such as social media are discussed in the literature, with parents’ expectations for treatments or surgeries stemming from other parents’ online experiences (Price and Mendizabal-Espinosa, 2019). Physicians and nurses highlighted unrealistic parent expectations as one of the three top barriers to their care delivery (Durall et al., 2012). This added complexity to staff experiences of providing care, as the same options are not available to all families and depend on the circumstances. Few papers have explored the influence of expectations and unmet needs in children’s palliative care services and studying barriers to children’s hospice and palliative care has also been recognised as a research priority (Baker et al., 2015).

As families will access various services during the child’s lifetime, the views of professionals from a range of these services must be harnessed in this type of research. A clearer understanding of the lived experiences of those professionals delivering care is warranted. Eliciting the views of these professionals and analysing the meaning of their lived experiences is a valuable way to understand how well they feel they can meet families’ needs, how families’ expectations influence the care they provide and how future care can be improved.

### Aim

To qualitatively explore professionals’ lived experiences of providing care to children with LLCs and their families.

## Method

### Design

This qualitative study, which adopted a phenomenological approach, sought to describe from the perspectives of professionals the essence of providing palliative care to families with children who have a life-limiting condition to unearth the meaning of their lived experiences (Teherani et al., 2015) of unmet needs, access and influence of expectations in CPC.

### Ethical approval

Ethical approval for the study was obtained from the Institute for Health Research Ethics Committee (IHREC), at University of Bedfordshire (Ref: IHREC851/IHRECC914)) on 13 June 2018.

### Population

Professionals were recruited from statutory and voluntary services at face-to-face events and advertisements were disseminated to relevant professional networks and special interest groups with the support of senior staff at Keech Hospice Care (KHC). Advertisements via posters, email and social network channels (e.g. Twitter and Facebook) were used, and snowballing techniques also facilitated recruitment to the study.

### Eligibility criteria

Professionals were eligible if they were currently working within a service which supports children with LLCs and their families, and this service was in and around Bedfordshire, Hertfordshire and Milton Keynes, UK. These geographic locations were chosen as they reflect the commissioned areas where KHC provides children’s services. It was beneficial to understand the experiences of those professionals working closely with one another to support families in these areas. Exclusion criteria included professionals who did not speak adequate English and those not actively working in this profession at the time of the interview.

### Procedure

Professionals received information about the study via face-to-face events, professional networks, special interest groups; posters; emails and social network channels (e.g., Twitter and Facebook).

Professionals who communicated an interest in participating were given a study invitation letter and participant information sheet with more detail. These professionals were screened in line with the pre-determined eligibility criteria. Professionals were then given time to consider the information and ask any questions they may have. Those who decided to participate were given a study consent form which they were asked to sign and return to the researcher. The researcher then arranged a convenient time for the interview.

Interviews were conducted between July and November 2018 and were held face-to-face or via telephone at a time and place convenient to participants, typically KHC or the participant’s place of work. Interviews were audio-recorded with participants' permission. The facilitator (PhD Candidate, GC) had extensive interviewing experience and ensured those who took part in this study were aware she was an independent researcher and not an employee of KHC to minimise bias. Interviews were carried out in a private room with the facilitator and participant.

A semi-structured interview schedule was developed following an extensive literature review and framed around findings of a scoping review of unmet needs which presented several critical areas of unmet need in CPC from the perspective of parents (Constantinou et al., 2019). As this study employed a phenomenological research approach, the researcher developed this schedule and carried out the research interviews wanting to understand the professionals’ lived experience of providing care and aimed to understand this phenomenon in a new light to make invisible aspects of the experience visible (Sundler et al., 2019). The schedule included open-ended questions used to allow participants to share; (1) lived experiences of effective children’s palliative care, (2) experiences of supporting families to access services and (3) their views on what an improved service would look like to them. The open-ended and semi-structured nature of the interviews allowed the participant to explore their experiences more freely and clarified the participant experiences with follow-up questions, which ensured the researcher understood the essence as intended by the participant.

After each interview, a trained transcriber transcribed the audio-recorded data into verbatim reports. These transcripts were reviewed by the researcher GC, to ensure the accuracy of the data. The researcher monitored the recruitment of professionals from various services for saturation of professional roles, while keeping a reflexive field diary of commonalities in the experiences being shared by professionals. Data collection was then stopped once the researcher felt saturation for professional roles and shared experiences had been reached, and no new experiences were felt to be drawn from the interviews.

### Data analysis

Transcripts were imported to NVIVO 12 software (QSR International Pty Ltd, 2018) and analysed following a thematic analysis informed by descriptive phenomenology (Sundler et al., 2019). This approach follows Braun and Clarke’s six-stage approach to thematic analysis (Braun and Clarke, 2006); the interconnected stages included; (1) familiarisation with the data, (2) generation of initial codes, (3) search for themes, (4) review themes and (5) define and name themes. While conducting this analysis, three crucial methodological principles of descriptive phenomenology: openness; questioning of pre-understanding; and adopting a reflexive attitude (Sundler et al., 2019) were followed to understand the meaning behind the lived experiences of professionals. An essential part of this process recognised the potential bias of the researcher when carrying out the data collection and analysis. This included previous experiences supporting families of children with LTCs and carrying out fieldwork in a hospice setting; therefore could be considered invested in this topic area. The researcher could be considered an ‘insider researcher’ (Greene, 2014), and this positionality bias was carefully questioned when interviewing and analysing data to ensure pre-determined frameworks of knowledge did not shape the meanings derived from the data. The researcher also ensured that the professionals recruited for the study knew that the interviewer was carrying out an independent study from KHC and was not an organisation employee, as this could potentially influence shared professional experiences. The first stage of open-minded reading was key to familiarisation with the data, ensuring the researcher was open to the text and its potential meanings. The initial thematic coding of the data was performed by the researcher (GC) to highlight meanings in relation to the study aims, the researcher made notes throughout this process. These codes were then discussed within the research team and reviewed by GC; themes were derived from patterns in the initial codes by GC and EC which were finalised in consultation with the second researcher (EC). The final code tree, code memos and coding were cross-referenced by a third independent researcher (GR) to ensure that the themes identified reflected the raw data. No changes were required. All key themes were cross-checked against the original data and coded quotes to ensure the reliability of the presented themes. Quotes were extracted to highlight specific themes.

## Findings

### Sample characteristics

A total of 29 professionals took part in the interviews (6 male; 23 female) who were representative of a range of relevant statutory and voluntary services (NICE, 2016). Participants shared experiences of supporting families in various settings, including; community care; intensive care; general and specialist hospitals; hospices; social care; general practice; education; therapy (complimentary, music, art, play, occupational, physio, speech and language); psychological/bereavement support and spiritualist services. Participants included; consultants, paediatricians, speciality doctors (hospice) (*n* = 5); nurses (IPU, ICU, community, clinical nurse specialists (CNS) (*n* = 5); general practitioners (*n* = 2); sisters/matrons (*n* = 3); healthcare assistants (*n* = 2); social care workers (*n* = 2); senior education staff (*n* = 1); occupational therapist (*n* = 1); play therapist (*n* = 1); complimentary therapists (*n* = 1); art and music therapists (*n* = 2); support workers (community, family) (*n* = 2); chaplaincy staff (*n* = 1) and a representative from a clinical commissioning group (*n* = 1).The majority of interviews were conducted face to face with participants, however, three participants chose to be interviewed via telephone methods. The duration of the interviews averaged 59.68 min with a standard deviation of 16.29.

### Thematic analysis informed by descriptive phenomenology

The findings from this study suggest three main themes: (1) Achieving effective care, (2) ‘These families don’t have time to wait’, and (3) ‘It moulds them into these people’. Several subthemes emerged within each theme, as shown in Table 1 below.

**Table 1.** Themes and subthemes identified from the professional experiences.

Themes	Subthemes
Achieving effective care	*‘A team around the child’*
	• Multi-disciplinary approaches; advance care planning; key working; family centred services; continuity with families
	*‘There’s not one way of documenting what’s going on with the child’*
	• Systems and communication
	*‘I don't want you to do it but I'm not going to’*
	• Working relationships; referral practices; lack of training; capacity of services.
‘These families don’t have time to wait’	*‘I wish that I could magic more staff out of thin air’*
	• Unfilled posts; lack of palliative care trained professionals; high turnover; finite resources; service closures; overstretched services; ‘postcode lottery’; financial concerns; distance to services
	*‘Reality is they don't get that, it's not possible’*
	• Sources of knowledge; need for support with knowledge gain; unrealistic expectations; comparison with other families.
	*‘They want to keep us at arm’s length for as long as possible’*
	• Stigma; fear; panic; resistance; misinterpretation; professionals’ personal views/attitude; awareness and education.
‘It moulds them into these people’	‘*They become quite militant’*
	• Fight and battle
	*‘I don't want to be in a service that you need to shout’*
	• Shout the loudest and suffer in silence
	*‘There’s this expectation that they have a bottomless resource to do what they do’*
	• 24-hour care; bottomless caring; sacrifice

#### Achieving effective care

Professionals described what it means to achieve effective care for families in many ways. Three subthemes were identified (1) *‘A team around the child’,* which detailed the approaches necessary for professionals to achieve the best care possible, (2) ‘There is not one way of documenting what’s going on with the child’ sharing experiences of systems and communication between services hindering the professionals care and (3) *‘I don’t want you to do it, but I’m not going to’* which pinpointed challenges working with other professionals, they had experienced while trying care for families.

#### ‘A team around the child’

Some viewed the importance of a multi-disciplinary team (MDT) working as crucial for effective care delivery. MDTs were viewed as a more efficient approach to care, limiting duplication, preserving resources and enabling more effective communication, relieving pressure on themselves and their families.

“if you've got a truly networked approach, particularly if somebody is approaching end of life not just their long-term holistic on going palliative care, you can take an awful lot of bump out of that by saying, you know - it's okay, we're in touch with your school, we're in touch with your statutory paediatrician, if you'll give consent we'll share information such that you don't always have to do that each time and to make good plans and really work as a team around the child.” (Paediatric Consultant)

Professionals also emphasised the value of advance care planning (ACP) to coordinate care, which they felt empowered them to respond to families appropriately. Clear ACP was argued to better coordinate care from various services reducing the need for parents' to have to retell their stories and giving professionals a clearer approach to proceed.

“If they have that [ACP) and it is coordinated well and they help hold on to that record, and with this coordinated approach to making sure that the services involved in that have a copy of that and the parent holds a copy as well. Then, in essence, wherever they go there would be a document, where they can go this is my child, I'm not going to tell you my story again this is what I have agreed with X, Y and Z professionals.” (Inpatient Unit Nurse)

Key workers and those working with families to coordinate care were also experienced as essential to successful care. Particularly if those key workers were experienced in supporting children with LLCs and had an understanding and connection to services, this was important for providing continued support.

“Having that type of key link, key professional, like one point of contact. It's somebody that needs to be like a key person that has a hand in all the services, that has direct links into each of the services that are available for these families to access, and it could access. Just to be one person and one service for a key point of contact, but then is able to support that family.” (Children's Community Nurse)

Family centred services that ‘wrap’ around the family and are shaped to that family’s individual needs were also deemed necessary. Professionals felt that continuity of care with families was vital to building trusting relationships. The high staff turnover represented a significant challenge to achieving this.

“When they have got a different social worker every three months, they can't do that; plus, they then have to dig up because when you get these social workers, they have to go through everything again and they have to dig up all their painful memories and stuff and tell someone new.” (Head Teacher)

#### ‘There’s not one way of documenting what’s going on with the child’

The shared experiences also showed challenging areas where professionals had found it difficult to meet the family’s needs. Communication within services and between the professional and family was raised as needing improvement. Language barriers: including the lack of availability of leaflets in a range of languages, concerns over informed decision making and advocating for children in these situations were raised.

“We've had families where the only people in a family that speak English are the children... if the parents don't speak English who's advocating them, you know, is it appropriate to be engaging a 12 year old in an end of life discussion about their sibling because the parents don't understand English?” (Senior Children's Nurse)

Miscommunication was also discussed to impact care delivery by wasting time through the need to chase for information. Many shared a lack of consistency in answering and returning calls as a frustration not just for themselves, but professionals also were concerned about the impact this can have on a family.

“you phone, you email, you don't get a response. And when you are a family doing that, that's really tough… it's bad enough when you're a professional it just gets on your nerves- when you're a family parents and you keep asking the same question and you're not getting a response it's very frustrating and adds to the pressure that you have already got,that you are already dealing with.” (Occupational Therapist).

How information is recorded and shared about the family with broader team members was raised by several professionals as an experience which made their role more difficult. Whilst services had their internal care records, these systems differed between services and could hinder the MDT team’s access to up-to-date information about the family, resulting in potential overlaps in care and a waste of resources. The need for a shared-care record system, accessible by all, was viewed as an important way of overcoming these barriers and would overcome the need for the responsibility to always fall on one professional, or detrimentally, the family.

“Some of that is that there is not one way of documenting what is going on with the child. There is no one system or process for doing that, that would depend on the service it involves.” (Children's Nurse)

Many professionals experienced this outside of the hospital setting:

“So we can't see what the hospitals are writing necessarily about patients, we're having to rely upon them sending us information or us chasing up information and I think that's a big problem… and as a result of that perhaps communication isn't as great and use different computer systems” (Social Worker).

However, professionals’ in the hospital itself also reported these experiences.

#### ‘I don’t want you to do it but I’m not going to’

Several issues were also raised concerning relationships between professionals and services, where some professionals work in isolation which could lead to mixed messages for the family. This protectiveness over roles and responsibilities was experienced by many professionals and indicated some experiences of negative attitude towards other services, which would impact the collaborative working approach that could be achieved:

“But there is still that kind of guarded 'I don't want you to do it but I'm not going to do it.' It needs to focus on what the family needs regardless of who is going to do it. I don't think it's a trust, I think it's a 'well it's not your area' does that make sense?” (Family Support Worker)

These experiences were frustrating for professionals and contrasted with the family centred approaches they raised as important in achieving effective care for families. Receiving late referrals was experienced by many professionals impacting relationships with the family and families’ trust. Experiences of working well as a team included providing consistent and clear messages to the family:

“to communicate together as a team and, and, and give a similar message to the family and the patient so there aren't mixed messages and to support them through the illness. Because the other thing I'm sort of aware of is that you know, conditions can change …I think the families can often get very confused, well certainly the mother going to see the different specialists and maybe getting different messages” (General Practitioner).

Frustration was also shared over the lack of referrals from certain professionals due to their attitude towards palliative care. Participants shared that whilst they could see areas where they could support a family, they often felt ‘blocked’ from doing so. This resulted in families potentially missing out on receiving extra support from other services which could contribute to meeting their needs:

“That can be very difficult and that can result in, you know, the family being reassured that they don't need the wider team they'll be, you know, 'I'll be able to look after all your needs, you don't need the need the hospice, no, no, you don't need to go there, of course you can stay at home' and for us that is a very frustrating feeling that actually we have been blocked out where we could have so much more [input].” (Consultant in Paediatric Palliative Medicine)

#### ‘These families don’t have time to wait’

Professionals explored the factors they experienced that influenced families’ ability to access the care they needed in a timely way. These experiences were developed into four subthemes; (1) ‘I wish that I could magic more staff out of thin air’ which discusses difficulties with reduced numbers of staff and overstretched services; (2) ‘Reality is they don’t get that, it’s not possible’ whereby experiences of sources of knowledge about the care that can be provided can inform unrealistic expectations in care and (3) ‘They want to keep us at arm’s length for as long as possible’ which discusses stigma, resistance and misinterpretation of services.

#### ‘I wish that I could magic more staff out of thin air’

Limited staffing, including unfilled nursing posts, limited numbers of palliative care trained paediatricians, high turnover, burnout, emotional impact, low pay and difficulties with recruitment were all frustrations that professionals shared experiences of. As one doctor states:

“*I wish that I could magic more staff out of thin air.”* (Speciality Doctor)

Frequent mention of funding being withdrawn, finite resources, closures of services due to lack of funds and overstretched services was also discussed in relation to the staffing issues raised.

“Lots of these centres that were able to deliver that kind of respite care to children are closing, funding is just not there for those types of services.” (Speciality Doctor)

These issues were also considered in the context of the ‘postcode lottery’, where funding limitations impact the availability of services. One occupational therapist discussed the variation of services available to parents from different geographical areas and explained the challenges faced by the professional to explain to families that they cannot access the exact services:

“*one family might be entitled to one package or something that looks like this, another family might not be assessed in the same way if they are from a different postcode, it's a postcode lottery.” (Occupational Therapist)*

Professionals shared frustration and feelings that this situation has got worse in recent times, limiting what care could be offered to families due to safety:

“We very nearly – a couple of weeks ago I couldn't fulfil a family's wish for palliative care because we didn't have enough staff. we have had to say - “no, sorry we can't do this because we don't have enough staff to safely deliver the care” - so we have been in that situation a couple of times over the year, more recently than in previous times” (Children's Community Nurse).

#### ‘Reality is they don’t get that, it’s not possible’

Professionals’ experiences of knowledge being sought by families from websites, online parent support groups and advice sought from other families were common. Professionals felt that knowledge and awareness of services was influential to access; with someone to provide correct information to families about what they may need to access and what is available, viewed as particularly beneficial to ensure families had realistic expectations of the care they could receive locally. Expectations were viewed to play a vital role in access to services through the way they perceive a service as to whether not it is suitable for them. Some professionals felt that parents’ perceptions were not static but dynamic and likely to change over time with exposure.

“I think depending on how the service has been sold as to what their expectations are, I think perhaps you'll find that the expectations that the family have in the beginning from the service to the expectations they have a couple of years down the line, might be quite different once they've become familiar.” (Children's Nurse)

Professionals experienced that families unfamiliar with services had limited expectations of them. However, those with more awareness, mainly when information had been sourced from parents, had somewhat higher expectations, which were sometimes unrealistic. Professionals were also concerned that other professionals who lacked experience in describing the service to a family might contribute to this expectation.

“I think the families, once they're at the door of the hospice and met the hospice I think very quickly it becomes more realistic…the expectations of it. I think it's just that initial referral, how that's managed. A deep anxiety around some professionals about what to offer and what to say is available.” (Children's Community Nurse)

#### ‘They…want to keep that at arm’s length for as long as possible’

These experiences of professionals’ not providing the correct information about the offer of a service at referral were related to experiences of the stigma associated with the word ‘hospice’ or ‘palliative’ care for the family, leading to difficulties on how to explain the care. Many professionals felt families viewed these services for death and dying rather than the broader opportunities for improvement in quality of life, meaning that the opportunity to support families is delayed. As one professional states:

“They have this idea of what a hospice is and want to keep that at arm's length for as long as possible and will only allow us in at the very last minute” (Community Nurse).

However, it was viewed that many who visit the hospice to look around often change their perceptions of and attitudes towards the service. Reluctance to refer patients due to professionals’ perceptions of and stigma towards palliative care services was also experienced and made meeting families’ needs difficult. One professional alludes to why this may be:

“because of the stigma over death, dying…going back to the stigma of, I think all; all doctors like to cure patients…and we don't like to say we've failed” (General Practitioner).

#### ‘It moulds them into these people’

Professional experiences supporting families to access care suggested families had specific approaches when interacting with professionals and often were firm in asserting their needs. This was disparate from how they felt families should interact with services. Three subthemes were identified; (1) ‘They become quite militant’, which detailed experiences of families assuming a role where they would fight against decisions that were made to secure what they need for their child (2) ‘I don’t want to be in a service that you need to shout’ which details experiences of interactions with families who either shout to be heard or suffer in silence and (3) ‘There’s this expectation that they have a bottomless resource to do what they do’ which emphasised professionals worry for how families would maintain 24-h care without proper short breaks available.

Professionals have witnessed many families having to ‘fight’ for things they need.

“Nearly all of these parents when you get them on a side situation, they will talk about having to fight. That’s the word that comes across all the time…I’m sure it’s true and they are not just fighting the diagnosis and the child’s illness they are fighting for the services they need in their home… proper mobility equipment, the right beds, the money the wet rooms all of those things which they need, just to live.” (Chaplain)

Caring for a child over a long time and experiencing resistance in accessing what they need was perceived to change parents’ behaviour to assume the ‘fighting’ stance. Professionals described interactions with families to reflect this:

I think some of our families that pick up some habits they become quite militant, the behaviour of the parents, but that's because they've had to fight for so long to get in within the NHS, whatever, and then they – yeah, I think it moulds them into these people that they probably weren't before they had their child…(Senior Children’s Nurse -P5).

#### ‘I don’t want to be in a service that you need to shout’

Professionals discussed parents’ response to the need to fight for things in two ways; several professionals used the phrase ‘shout the loudest’ to describe parent’s behaviour to obtain what they need for their children. As one social worker states:

*“parents are very much becoming of the culture that if they shout, they will get. I don't want to be in a service that you need to shout, we should be robust in our practice where parents will be – they don't need to shout.*” *(Social Worker)*

Alongside these experiences’ professionals showed concern for other families who ‘suffer in silence’ and do not make professionals aware they need support.

“Others I think just suffer in silence and don’t say anything, they have to, they have to just cope” (Occupational Therapist)

Professionals felt that change needed to happen to protect those parents who are not being heard by services:

“I think it's time to listen out for the people who don't have a voice, versus always responding to people who do” (Supportive Care Therapist).

#### ‘There’s this expectation that they have a bottomless resource to do what they do’

Professionals also experienced families providing 24-h care. This was a worry for many professionals, whereby without regular relief from caring, the well-being of these parents and the emotional impact is detrimental.

“When we're talking about an ill child, they're ill 24 hours a day, they're not ill between the hours of 9 till 5.” (Art Therapist)

Professionals also expressed concern for the safety of the parent and the child in these situations.

“she's had a few bad nights and Mum just looked awful and I was quite worried, and I was also worried for the child.” (Clinical Nurse Specialist)

Professionals feared that this ability to care is viewed as a ‘bottomless resource’ which, if left with no break from their caring role, could lead to a crisis.

“There is this expectation that they've got a bottomless resource to do what they do.”(Complimentary Therapist)

It was also discussed that to fulfil this responsibility, parents made sacrifices, including: time with siblings; mental health; sleep; and ability to work. More investment in offering family respite was therefore viewed as particularly important.

“But I think it’s kind of short sighted because, if we invested more in helping families erm to care for their children who are at home and have proper respite we wouldn’t have as many crises as we end up getting.” (Speciality Doctor)

## Discussion

This study aimed to examine professionals’ experiences of providing care to children with LLCs and their families to understand the unmet needs, access issues and influence of expectations in CPC. This study used semi-structured interviews with professionals from a range of CPC services to achieve this aim. This paper reports the findings derived from the interviews, which included (1) ‘achieving effective care’; (2) ‘these families don’t have time to wait’; and (3) ‘it moulds them into these people’.

Experiences indicated unmet needs surrounding communication in care with: miscommunication; lack of information; and lack of consistency in answering/returning calls, cited as barriers to meeting the family’s needs. A review of the existing literature evidences communication to be a particular unmet need for families internationally (Constantinou et al., 2019). For professionals in this study, language barriers were also present in some of their consultations, with difficulties surrounding informed choice and advocating for children when these are present. As the study was carried out in Luton, a town in Bedfordshire in the UK known for its culturally diverse population (Luton Borough Council, 2011), it is not surprising that professionals’ experiences reflect this. It is known that this group has been less likely to come forward to access palliative care (Gatrad et al., 2008), therefore, emphasising why tailored resources to have conversations about care with the family are so important to ensure they are cared for appropriately and are aware of their choices. Using professional interpreters is suggested to be helpful, but it is recognised that the logistics of tapping into these resources can be problematic (Ali and Watson, 2018).

A crucial part of achieving effective care was influenced by how professionals from the various services were able to work with one another. Whilst professionals appeared aware of the approaches and skills needed to deliver care effectively with other services such as: advance care planning; multi-disciplinary team working; and networked approaches (NICE, 2016), experiences suggested this was not always standard practice or being facilitated readily enough. The use of managed clinical networks (MCN) aims to improve the coordination of palliative care and facilitate effective working between services irrespective of professional boundaries (Papworth et al., 2021). However, the professional relationships were strained in some experiences, and a perceived ‘overprotectiveness’ was revealed in guarding families, roles and responsibilities. This was experienced to inhibit professional access to certain families through referrals to hospice care or palliative care teams, which left them unable to meet their needs appropriately. Existing evidence shows the influence of stigma relating to palliative care as a barrier to achieving referrals, with fear from patients, professionals and families impacting families’ choices in care (Sayma et al., 2020). A review of service providers’ and families’ perspectives of barriers to accessing specialist palliative care also found uncertainty about what this type of care offers and a clear association between palliative care as EOL care (Taylor et al., 2020). These issues, therefore, may explain why relationships can be strained in some cases and stress the importance of strengthening the links between services and addressing misunderstandings as to who may benefit from CPC services, which is recognised to be an issue (El-Jawahri et al., 2017). It is essential to note that the benefits of this care are often overshadowed by the stigmatisation attached to the phrase (Boyd et al., 2019).

While findings of protectiveness over roles, responsibilities and consequently family referrals could be due to a lack of networked relationships and understanding of CPC, it is also suggested that families have pre-established trusting relationships with certain professionals (Gómez-Zúñiga et al., 2019) and are keen to maintain continuity which may explain why some families are more reluctant to be referred. Evidence also suggests that families accessing hospice care services may require time to build a sense of ‘belongingness’ with the place in question (Dunbar and Carter, 2021). It could be this unfamiliarity with the service or professional which creates resistance by the family and, therefore, a perceived protectiveness over the referral of families, which could be the families’ reluctance to access a new professional’s support.

Services often used internal recording systems for information about the family that were not consistent with other services. This was experienced to lead to duplication of work, a waste of resources and a lack of clarity of the families’ up-to-date circumstances. Professionals’ stressed the need for a shared-care record which would allow them to work more effectively. Families have previously reported experiences of having to retell their stories due to this issue (Hunt et al., 2013; Whiting, 2013). Without transparent systems between services, the responsibility can often fall on the ‘care coordinator’ to manage enquiries related to the child and share information between professionals (Hillis et al., 2016), however, how well this role is performed remains unclear. Having shared technology between services supporting the family would be beneficial to facilitate the networking between services (Papworth et al., 2021) but currently this is an unresolved barrier facing professionals.

The findings revealed a wide range of factors that influenced access to palliative care services. Professionals’ experiences of feeling ‘blocked’ by other professionals from accessing a family and providing care were shown to be a result of the impact of misunderstandings of CPC. These experiences were closely related to expectations of care, with some referrals made being portrayed to the family to offer them unrealistic care. This was thought to result from the referring professionals ‘overselling’ of the hospice service due to their concerns about how the family may react. This made it difficult for professionals to revaluate expectations with families to ensure they were more realistic about what could be provided. This reinforces the importance of education and awareness of hospices and palliative care in reducing stigma and ensuring they are aware of appropriate information for the families and professionals from other services. Parents’ expectations were also influenced by information sought from other parents, social media and online research, with professionals finding higher expectations in those who were more connected. As the literature suggests that not knowing what to expect from their child’s care can reduce feelings of control over their situation (Verberne et al., 2019), being informed helps them determine what to expect from services which is a crucial support need for parents (Aoun et al., 2020), even if these are not always achievable.

Families’ interactions with professionals made providing care challenging in some instances. Professionals had witnessed a change in approach from some families, describing them as becoming ‘militant’ to secure what their child needed because of previous resistance. The reality of caring on a day-to-day basis that parents have to uphold was discussed to lead to a stance of needing to ‘fight’ and battle’ for what they needed, this was a widely shared experience with professionals, supporting previous research conducted over ten years ago, where families were reported to be ‘fighting for care’, ‘battling’ and ‘jumping through hoops’(Darbyshire et al., 2015; Doig et al., 2009; Whiting, 2013). More recently, families are still described to be fighting the system and feeling unheard (Mitchell et al., 2021), a disappointing contrast to the guidelines for CPC recommending choice (Together for Short Lives, 2018). The current findings suggested that some parents interacted with professionals in one of two ways; those who (a) shout the loudest and those who (b) suffer in silence. Professionals felt that those who ‘shout the loudest’ could create difficulty with ‘unrealistic’ demands and overshadow those parents perceived to ‘suffer in silence’ who were viewed as not getting the attention they deserve. Professionals did not want to be in a role which led to these interactions being common and stressed that families need better access to short breaks from caring to prevent these experiences.

### Strengths and limitations

The importance of uncovering professionals’ experiences in providing palliative care is pivotal to improving the quality of provision for children with LLCs and their families. This study contributes to the national and international research priorities (Baker et al., 2015; Downing et al., 2015) by identifying the issues professionals face and the circumstances in which they arise, a gap in the evidence base (Together for Short Lives, 2018). The qualitative approach in this study allows for in-depth exploration of professionals’ experiences and detailed accounts of what it is like to provide care to families. Through an inclusive approach to recruitment, this study was able to capture the experiences of professionals who worked across a range of voluntary and statutory services responsible for providing palliative care to families of children with LLCs, a strength of the study. Whilst a wide range of roles were recruited, there were notable challenges in recruiting professionals from respite, psychological and mental health and speech and language provisions to participate. These areas are known to have wait times and challenges in access for families and were areas outlined by the professionals in this study as areas of concern. The study population was relatively small, interviewing 29 professionals caring for families in Bedfordshire, Hertfordshire and Milton Keynes, so their views may not be transferrable to a broader population, however, many of the principles will still stand.

### Implications for practice

Professionals’ experiences detail examples of good quality care for the families they support, such as MDT, key working, ACP and networking, emphasising the importance of implementing these approaches to meeting families’ needs. Experiences also showed areas in which further attention was required to improve care at present. The findings can be incorporated into professionals practice to better support the families they are working with, such as identifying those families who may be ‘suffering in silence’ and not asking for the additional support they need and working closely with other services to identify procedures for collaborating to support families more effectively. Further research is required to understand the barriers to collaborative working in CPC, particularly understanding which service issues arise to reduce service fragmentation. Professionals’ understanding and parents’ awareness of services is needed to inform their expectations of care, mainly if this information was sourced online or from other families. Services should identify if families are being provided with the necessary information to form an appropriate expectation of available services. These perspectives share meaningful first-hand experiences of unmet needs, access and expectations of palliative care and have the potential to inform future policy development and service design.

## Conclusion

This study provides valuable insight into what it means to professionals to provide CPC through interviews with various professionals working with families. The findings suggest that professionals are working hard to deliver care while experiencing families’ ‘fighting’ to receive support. Experiences of dealing with parents who need to ‘shout the loudest’ to be heard are common and frustrating for professionals and not the service they envision. An identified need for improved collaborative working between services, awareness and education surrounding hospices and palliative care and fears about the parent’s responsibility to provide 24 h care over extended periods was shared, with concern about parents being used as a ‘bottomless’ caring resource leading to a crisis if not provided a break from their caring role.
